# The prevalence of reasons for tooth extraction in cats

**DOI:** 10.3389/fvets.2025.1626701

**Published:** 2025-07-14

**Authors:** Chun-Geun Kim, Daehyun Kwon, Kyuyoung Lee, Se Eun Kim, Hyun Min Jo

**Affiliations:** ^1^Evichi Veterinary Dental Hospital, Seoul, Republic of Korea; ^2^May Veterinary Dental Hospital, Seoul, Republic of Korea; ^3^Department of Veterinary Surgery, College of Veterinary Medicine and BK21 Plus Project Team, Chonnam National University, Gwangju, Republic of Korea; ^4^Department of Microbiology, Institute for Viral Diseases, College of Medicine, Korea University, Seoul, Republic of Korea; ^5^Biomaterial R&BD Center, Chonnam National University, Gwangju, Republic of Korea

**Keywords:** oral disease, tooth extraction, periodontitis, FCGS, TR, feline

## Abstract

**Objectives:**

This study aimed to determine the prevalence of diseases leading to tooth extraction in a large population of cats and to identify factors associated with each condition, including age, sex, breed, and tooth position.

**Methods:**

All cats underwent a thorough dental examination and full mouth dental radiographs to assess their oral health. Each tooth was classified according to its primary pathological condition, and extraction was performed based on clinical criteria specific to each disease. Tooth extractions were then performed using appropriate methods based on the condition of each tooth. For a detailed analysis of each disease, a comparative study was conducted considering factors such as sex, age, breed, and tooth position.

**Results:**

Periodontitis (33.41%), feline chronic gingivostomatitis (FCGS, 32.40%), and tooth resorption (TR, 15.21%) were the three most frequent reasons for tooth extraction in cats. Periodontitis and TR tended to significantly increase in prevalence with age, while FCGS showed a decreasing trend with age. Periodontitis was most commonly observed in the maxillary first molar teeth and mandibular incisor teeth, and least frequently in the canine teeth, with no statistically significant differences by sex or breed. FCGS showed a higher prevalence in domestic shorthair cats, and a slightly higher extraction rate in intact females compared to spayed females. There were no statistically significant differences by tooth location. TR was most frequently found in the mandibular third premolar teeth, with no significant variation by sex. While some breeds showed higher TR prevalence, the distribution varied across breeds.

**Conclusions and relevance:**

This study underscores the importance of tailored dental care for cats, especially as they age, and highlights the need for further studies to explore the links between factors such as nutrition, genetics, and oral health. A better understanding of these aspects can lead to improved overall health and quality of life for cats.

## 1 Introduction

With the recent growing interest in and importance of veterinary dentistry, diagnosing and treating oral diseases have become important research topics in the health management of companion animals. Dental disease is highly prevalent in companion animals, with studies reporting that over 70% of cats exhibit some form of dental pathology (1, 2). According to large-scale primary-care studies, dental conditions accounted for approximately 12.5% of diagnoses in dogs and 15% in cats (3, 4). These figures reflect the proportion of dental cases among animals presenting to veterinary hospitals and demonstrate the clinical relevance of dental disease in companion animals. Since dental diseases are not easily recognized through visual examination in the early stages, accurate evaluation through dental examination and dental radiography under anesthesia is often required (5).

Despite their clinical importance, feline dental diseases have received less research attention compared to those in dogs. Cats are sensitive to stress and pain, and even mild oral diseases can lead to loss of appetite and decreased quality of life. In addition, chronic oral inflammation has been reported to contribute to systemic consequences (6). However, due to the characteristics of cats, regular oral care, particularly home care such as tooth brushing, is often challenging (7), making it difficult for owners to observe the inside of their cat’s mouth. As a result, early-stage dental problems often go unnoticed, and treatment is frequently delayed until the disease becomes more advanced (6). Therefore, there is a need for more detailed studies that consider the unique behavioral and anatomical characteristics of cats.

Common diseases in cats include tooth resorption (TR) and feline chronic gingivostomatitis (FCGS) (8, 9). TR is characterized by progressive loss of dental hard tissue (cementum, enamel, and/or dentin) due to odontoclastic activity, and in some cases, may be accompanied by changes in the surrounding periodontal tissues. The exact cause of TR has not yet been identified, but this condition is one of the most common diseases in cats, and according to several studies, it accounts for 28.5–67% of oral diseases (10). FCGS is a disease that causes chronic inflammation of the oral gingiva and mucosa, causing severe pain and a decrease in quality of life (11). In severe cases, it can significantly impair quality of life and may even lead to considerations for euthanasia (12). It is also a common disease in cats, with a reported prevalence rate of up to 26% in domestic cats (13). In addition, various diseases such as periodontitis and endodontic disease can occur, and the most common treatment method for these conditions, as in dogs, is tooth extraction.

We hypothesized that the prevalence and distribution of dental diseases requiring extraction in cats would vary significantly by age, breed, sex, and tooth location. Therefore, this retrospective study aims to analyze the occurrence of dental diseases according to sex, age, breed, and tooth location in cats, and to classify these diseases based on the frequency with which extraction was performed as the primary treatment, reflecting actual clinical practice. Furthermore, this study aims to evaluate the primary dental diseases leading to tooth extraction in cats and how these conditions vary across different biological and anatomical parameters.

## 2 Materials and methods

### 2.1 Case inclusion

Between January 2015 and June 2021, 1,580 client-owned cats were seen for dental examinations and treatment. Evichi Veterinary Dental Hospital (EVDH, CGK) received 958 of these cats, and May Veterinary Dental Hospital (MVDH, D-HK) received 622.

Only cats that had complete dental charting and full-mouth intraoral radiographs performed, and at least one permanent tooth extracted were included in this study. Cats who underwent only professional teeth cleaning or extraction of persistent deciduous teeth were excluded.

For breed-based analysis, breeds with a population of more than 50 cats were classified as major breeds, while those with fewer than 50 cats were classified as minor breeds.

### 2.2 Medical record review

As in previous research (14), each cat’s information including their signalment history, physical examination findings, laboratory diagnostics, diagnostic imaging results, oral pictures, and dental charts was obtained from their electronic medical records.

### 2.3 Intraoral radiography

A full-mouth radiography examination was performed on all the cats utilizing a standardized system of positioning, views, and techniques. The radiographs were taken with a standard wall-mounted dental radiography unit (Progeny® Dental, Midmark, Seoul, Korea) equipped with a photostimulable phosphor plate (PSP) system (CR7VET, iM3, SHINKI, Seoul, Korea) with size no. 0–4 plates and a direct digital imaging system with a no. 2 sensor (EVA-VET; AFP Imaging, Elmsford, NY). Each veterinarian (C-GK and D-HK) performed the interpretation of the radiographs.

### 2.4 Classification of the tooth resorption

The classification of tooth resorption was based on the American veterinary dental college (AVDC) nomenclature (15), which categorized it according to severity (stages) and location (types). In this study, tooth resorption was defined as the loss of dental hard tissue (enamel, dentin, and/or cementum) due to odontoclastic activity. Cases of root resorption secondary to periodontitis or endodontic infection were classified separately as inflammatory root resorption and excluded from the analysis of the primary cause of tooth resorption.

### 2.5 Oral examination and dental chart recording

C-GK and D-HK conducted systematic oral examinations, including the assessment of plaque and calculus index (16), gingival index (17), stages of tooth mobility (18), and stages of furcation involvement (18), with all patient positioned identically. Skilled technicians documented the results of these examinations.

### 2.6 Classification of causes for extractions

All cats were grouped in the study according to their breed, age, sex, and tooth position. A total of 47,400 teeth from 1,580 cats were categorized into untreated, missing, and extracted groups to describe the overall dental status, as complete dentition was uncommon in clinical cases. However, only the extracted teeth were used for statistical analysis in this study. For a more thorough examination, the diseases related to tooth extractions were further divided into 14 different categories within the subset of extracted teeth. The names of various pathology that resulted in tooth extractions and the abbreviations that correlate with them are shown in the following table (Table 1). The TF category was composed of four types defined by the AVDC nomenclature (15): uncomplicated crown fractures (with periapical lesions and/or failure to narrow of the pulp cavity), root fractures, complicated crown-root fractures, and complicated crown fractures.

**Table 1:** Abbreviations for various oral diseases.

No.	Abbreviations	Full text
1	FN	Failure to narrow of the pulp cavity
2	MAL	Malocclusion
3	OT	Oral tumor-engulfed teeth
4	P	Periodontitis
5	PA	Periodontitis with alveolar bone expansion
6	PATR	Periodontitis with alveolar bone expansion and tooth resorption
7	PAIRR	Periodontitis with alveolar bone expansion and inflammatory root resorption
8	PTR	Periodontitis with tooth resorption
9	PIRR	Periodontitis with inflammatory root resorption
10	RTR	Retained root tip
11	FCGS	Feline chronic gingivostomatitis
12	T/SN	Supernumerary teeth with periodontitis
13	TR	Tooth resorption
14	T/FX	Tooth fracture

### 2.7 Criteria for tooth extractions

Tooth extractions were performed based on specific clinical and radiographic criteria corresponding to each disease category. These criteria included factors such as tooth vitality, presence of periodontal or endodontic disease, structural damage, and treatment feasibility. A detailed summary of the extraction indications for each condition is provided in Table 2.

**Table 2:** Criteria for tooth extraction by disease category.

No.	Abbreviations	Full text
1	FN	No evidence of a crown or root fracture (if endodontic treatment is not an option)
2	MAL	Malpositioned teeth are injuring soft tissue or other teeth (if orthodontic movement is not an option)
3	OT	In the process of the tumor removal
4	P	Irreversible damage from severe periodontal disease (when at least one of the following is present: mobility of stage II or greater with alveolar bone loss exceeding 50%, or stage III furcation involvement)
5	PA	Follows the extraction criteria for P
6	PATR	Follows the extraction criteria for P and TR
7	PAIRR	Follows the extraction criteria for P and root resorption
8	PTR	Follows the extraction criteria for P and TR
9	PIRR	Follows the extraction criteria for P and root resorption
10	RTR	Retained root tips due to tooth fracture, and retained root tips associated with tooth resorption showing a visible periodontal ligament space and root canal on dental radiographs
11	FCGS	Severe or ulcerative gingivitis and stomatitis. Partial mouth extraction (extraction of all premolar and molar teeth): when caudal inflammation was present. Full-mouth extraction (extraction of all teeth): in cases with both rostral and caudal inflammation
12	T/SN	Supernumerary teeth associated with periodontitis
13	TR	All types of tooth resorption. In cases consistent with type 2 or type 3 resorption^※^, coronectomy may be considered based on radiographic findings.
14	T/FX	Non-vital teeth or fractured teeth with pulp exposure (if endodontic treatment is not an option due to the severity of the tooth fracture, presence of root pathology, the cat’s overall health condition, or the owner’s preference)

^**※**^Coronectomy: In cases where radiographic findings were consistent with type 2 or type 3 resorption without evidence of periodontal or endodontic lesions, the condition was characterized by narrowing or loss of the periodontal ligament space in specific areas and decreased radiopacity in portions of the tooth.

### 2.8 Tooth extraction methods

All cats were anesthetized, induced with injectable medication, and maintained under inhalational anesthesia with continuous monitoring. Detailed anesthetic protocols are beyond the scope of this study. The appropriate instruments, equipment, and surgical methods were used for tooth extraction (19–23). Depending on the pathology of the tooth, alveolar bone condition, and anatomical characteristics, most single-rooted teeth were removed by simple extraction, whereas multi-rooted teeth and single-rooted canine teeth were typically extracted using surgical extraction. The general purpose of tooth extraction was to eliminate all the tooth roots. Based on dental radiographic examination, a coronectomy (intentional root retention) or complete extraction was performed as appropriate for the type and stage of tooth resorption. After complete root removal, alveoplasty was carried out with a round diamond bur on a high-speed handpiece to smooth any sharp bony margins. Flaps were raised and sutured without tension using a 5–0 poliglecaprone 25 synthetic absorbable monofilament suture after inflammatory tissue was removed from all extraction sites. Postoperative radiographic evaluation was performed when the extracted tooth exhibited sharp root morphology or when a root fracture was suspected.

### 2.9 Statistical analysis

The present study summarized descriptive statistics of oral diseases in cats utilizing frequency tables and graphs based on data from electronic medical records. Non-parametric one-way analysis of variance (Kruskal–Wallis test) was used to evaluate the association of the number of teeth with periodontitis, FCGS, or TR with demographic factors considering the violation of normality assumption. Pairwise Wilcoxon rank sum tests were conducted to compare the distribution of the number of teeth with periodontitis, FCGS, or TR by each pair of groups of a demographic factor as the *post-hoc* test of the Kruskal–Wallis test. All data management, statistical analyses, and visualization were performed with R studio version (4.4.1; R Core Team). Our study utilized a cut-off *p* < 0.05 to determine statistical significance. Our study used the Shapiro–Wilk test to assess the normality of the distributions for the number of extracted teeth and age (*p* < 0.05).

## 3 Results

### 3.1 Proportion of cats and extracted teeth among 1,580 felines based on different classifications

#### 3.1.1 Proportion of cats by sex, age, and breed

**Figure 1:**
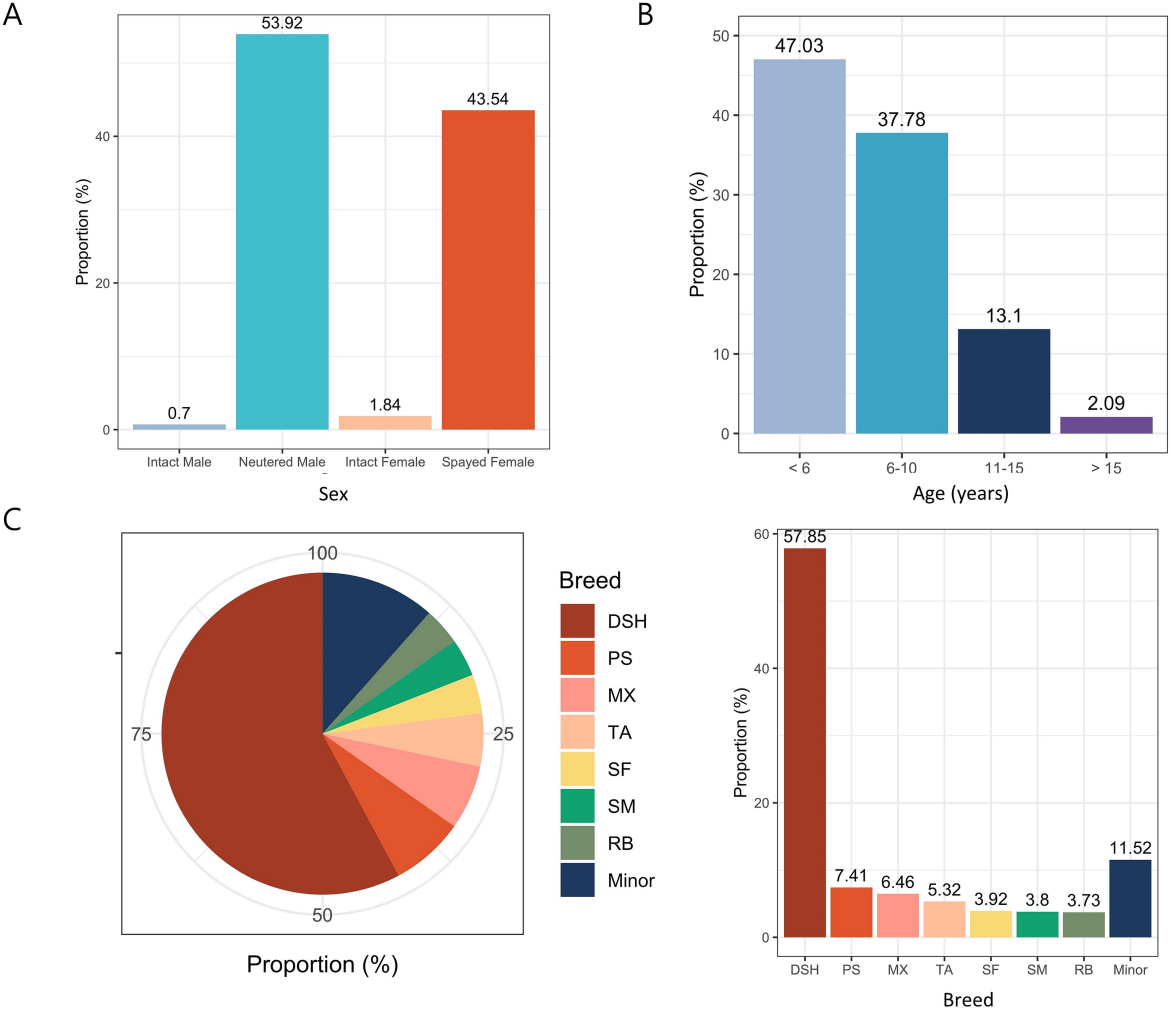
Proportions of 1,580 cats according to various classifications. **(A)** Sex distribution. **(B)** Age distribution. **(C)** Breed distribution. DSH, Domestic shorthair; PS, Persian; MX, Mixed; TA, Turkish Angora; SF, Scottish Fold; SM, Siamese; RB, Russian Blue.

To investigate the factors that lead to tooth extraction, this study thoroughly analyzed 1,580 cats of 24 different breeds and focused on the effects of sex, age, and breed. In terms of sex, there were 0.70% (11/1,580) intact males, 1.84% (29/1,580) intact females, 43.54% (688/1,580) spayed females, and 53.92% (852/1,580) neutered males. Interestingly, there were significantly more spayed/neutered cats (97.5%) than intact cats (2.5%) (Figure 1A). In terms of age, most cats were aged <6 years (Group 1: 47.03%, 743/1,580), followed by cats aged 6–10 years (Group 2: 37.78%, 597/1,580), those aged 11–15 years (Group 3: 13.10%, 207/1,580), and those aged >15 years (Group 4: 2.09%, 33/1,580) (Figure 1B). Domestic shorthair, Persian, mixed breeds, Turkish Angora, Scottish Fold, Siamese, and Russian Blue were among the most common breeds (Figure 1C). Minor breeds were defined as those represented by <50 cats.

#### 3.1.2 Distribution of untreated, extracted, and missing teeth in 1,580 cats

**Figure 2:**
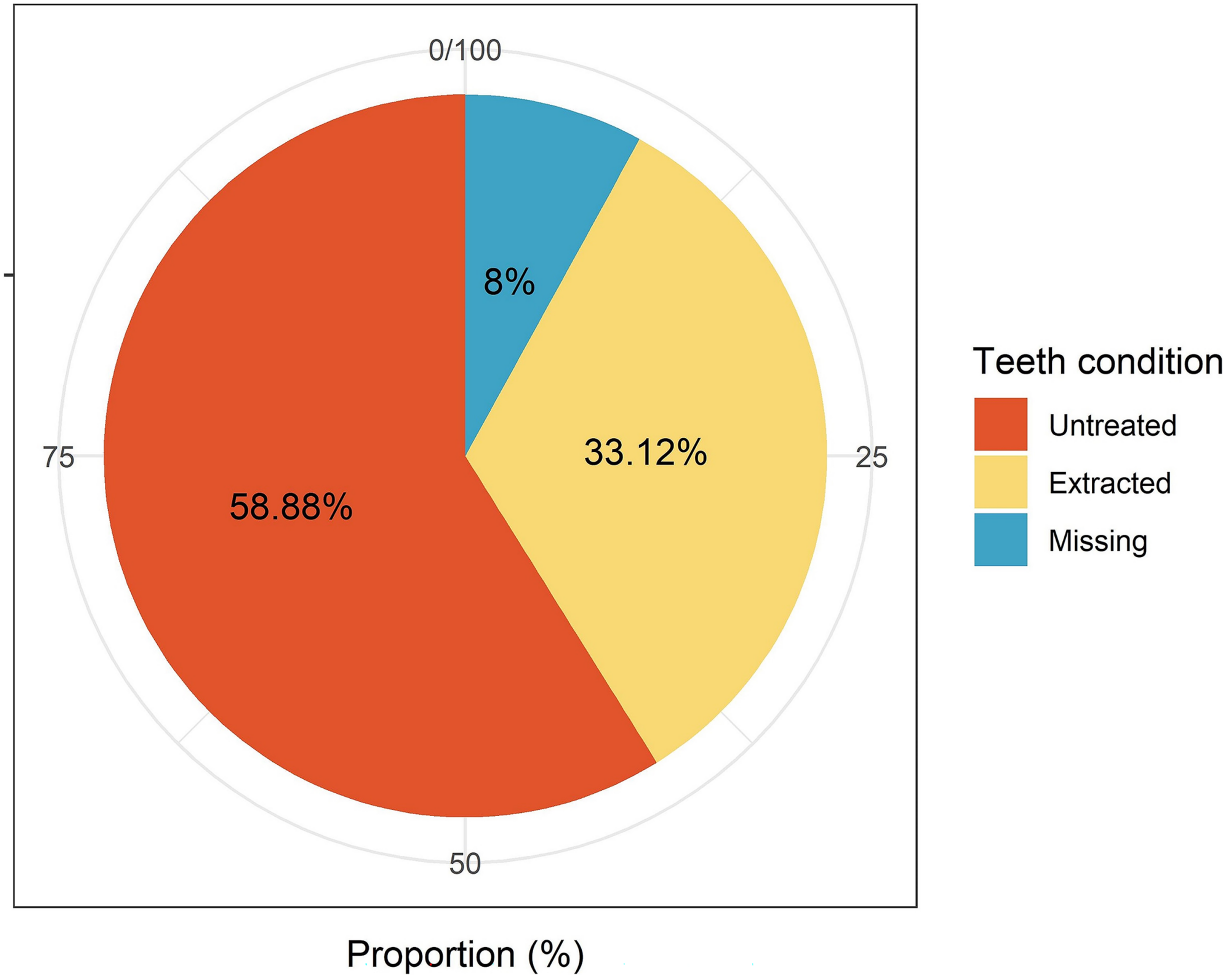
Distribution of untreated, extracted, and missing teeth.

Through a comprehensive examination in the study, 47,400 possible permanent teeth from 1,580 cats were evaluated. Among these, 33.12% were extracted, 8.00% were missing, and 58.88% did not require extraction (Figure 2).

#### 3.1.3 Proportions of various oral diseases leading to tooth extractions

**Figure 3:**
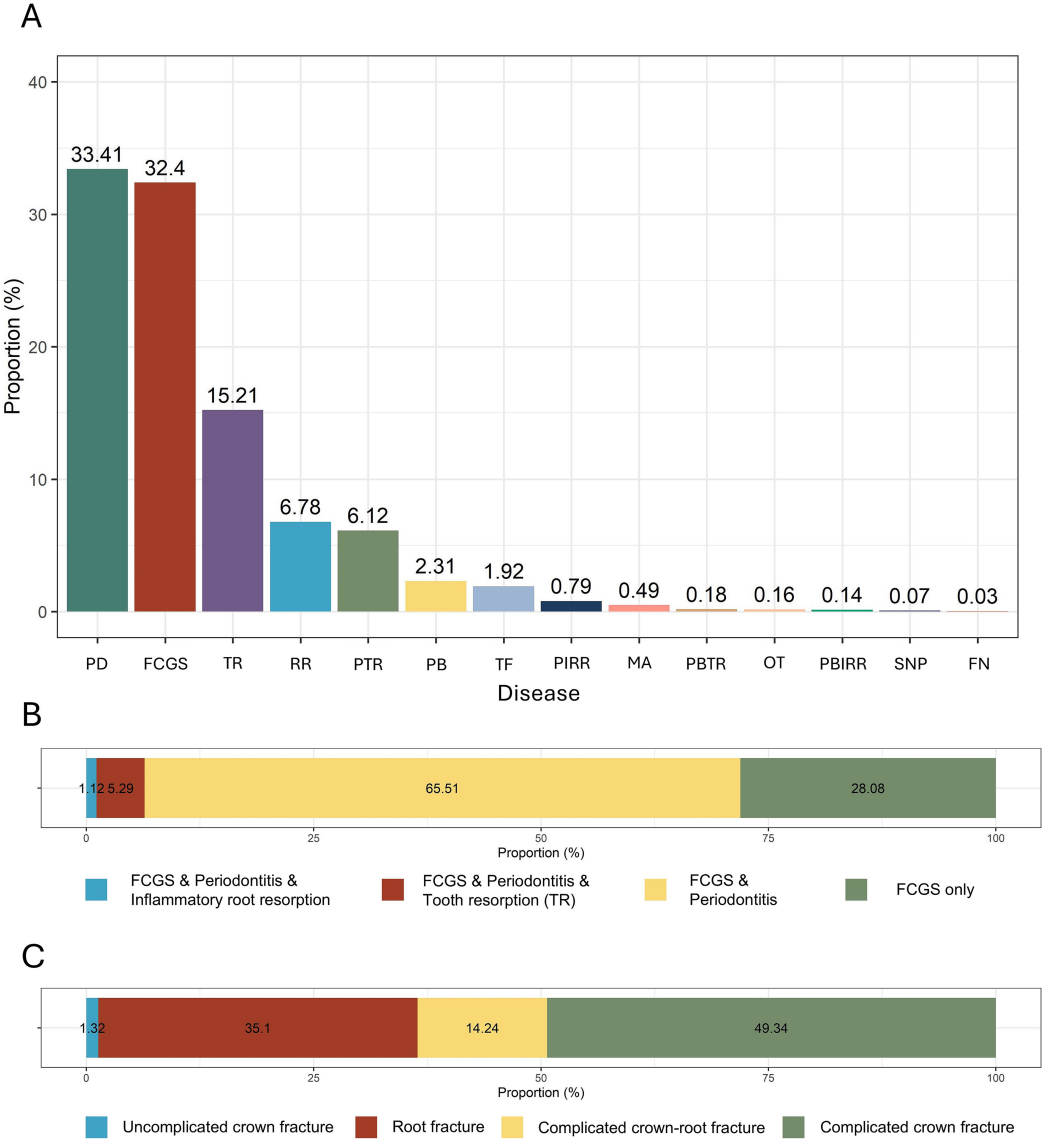
Overview of the proportion of various oral diseases leading to tooth extraction. **(A)** Proportions of causes of tooth extraction by each oral disease. **(B)** Classification of FCGS based on the presence of periodontitis and/or tooth resorption. **(C)** Proportions of different types of tooth fractures causing tooth extractions. P, Periodontitis; FCGS, feline chronic gingivostomatitis; TR, tooth resorption; RTR, retained root tip; PTR, periodontitis and tooth resorption; PA, periodontal disease and alveolar bone expansion; T/FX, tooth fracture; PIRR, periodontitis and inflammatory root resorption; MAL, malocclusion; PATR, periodontitis and alveolar bone expansion and tooth resorption; OT, oral tumor; PAIRR; periodontitis and alveolar bone expansion and inflammatory root resorption; T/SN, supernumerary and periodontitis; FN, failure to narrow of the pulp cavity.

Periodontitis accounted for the largest proportion at 33.41%, followed by FCGS and TR at 32.40 and 15.21%, respectively. The top three diseases were considered major causes of tooth extraction, and other oral diseases were classified as minor diseases (Figure 3A). In addition, FCGS was classified into four types again according to whether it was accompanied by periodontitis and/or tooth resorption, inflammatory root resorption, and the results showed that FCGS and periodontitis occurred simultaneously in 65.51% of cases and that FCGS occurred alone in 28.08% of cases (Figure 3B). Among the tooth extractions performed due to fractures, complicated crown fractures accounted for the highest proportion (49.34%), followed by root fractures (35.10%), complicated crown-root fractures (14.24%), and uncomplicated crown fractures (1.32%) (Figure 3C).

#### 3.1.4 Distribution of various oral diseases in each tooth among the maxillary and mandibular extracted teeth in 1,580 cats

A tooth-by-tooth analysis confirmed that periodontitis, FCGS, and tooth resorption remained the most common causes of extraction across individual teeth, consistent with the overall trend observed in the population-level summary. In addition, TR was especially prevalent in the mandibular third premolar teeth, with rates of 45.4% (Left) and 45.2% (Right). Retained roots showed a high rate in incisor teeth in both the maxilla and mandible. In addition, tooth fracture was observed most commonly in canine teeth, with 14.8% in the maxillary right canine and 14.9% in the maxillary left canine teeth (Tables 3, 4).

**Table 3:** Prevalence of oral diseases in each tooth among maxillary extracted teeth.

Categories		Prevalence of oral diseases (%)															
		Right maxilla								Left maxilla							
		M1	P4	P3	P2	C	I3	I2	I1	I1	I2	I3	C	P2	P3	P4	M1
Number of extracted teeth		621	603	646	611	443	447	413	412	410	414	420	447	608	631	614	637
FN		0.0	0.0	0.0	0.0	0.2	0.0	0.0	0.0	0.0	0.0	0.2	0.2	0.0	0.0	0.2	0.0
MAL		0.0	3.0	0.9	0.0	0.7	0.0	0.0	0.0	0.0	0.0	0.0	1.3	0.0	1.0	3.6	0.0
OT		0.0	0.0	0.0	0.0	0.5	0.4	0.5	0.2	0.2	0.2	0.2	0.0	0.2	0.0	0.2	0.2
P		58.9	38.8	28.2	34.0	18.7	47.7	44.6	41.3	41.0	44.0	41.9	19.7	31.1	25.5	36.6	57.1
PA		3.2	3.5	3.3	2.1	8.1	0.0	0.0	0.0	0.0	0.0	0.0	8.7	2.6	3.8	3.7	3.0
PATR		0.2	0.3	0.2	0.0	0.0	0.0	0.0	0.0	0.0	0.0	0.0	0.7	0.3	0.5	0.7	0.3
PAIRR		0.0	0.0	0.0	0.0	0.5	0.0	0.0	0.0	0.0	0.0	0.0	0.4	0.0	0.0	0.0	0.0
PTR		1.4	7.8	9.9	2.9	6.3	0.7	1.7	1.5	1.2	1.0	1.2	4.9	3.0	10.8	7.0	1.1
PIRR		0.0	0.8	0.3	0.0	0.5	0.7	0.2	0.0	0.0	0.2	0.2	0.2	0.0	0.3	0.8	0.0
RTR		1.8	3.3	2.5	3.8	1.4	13.2	11.9	16.5	15.4	13.3	14.5	2.0	3.1	3.0	4.2	2.8
FCGS	S	6.0	8.1	9.4	9.7	11.3	11.0	12.8	12.6	6.0	8.1	9.4	9.7	11.3	11.0	12.8	12.6
	SP	24.3	27.0	21.5	15.1	19.2	20.4	22.3	21.1	22.4	23.2	23.3	20.4	17.1	22.0	26.7	24.8
	SPTR	0.5	1.2	2.6	1.6	0.5	0.4	0.0	0.0	0.7	0.7	0.2	0.7	1.0	2.2	1.1	0.3
	SPIRR	0.0	0.0	0.0	0.0	0.2	0.0	0.0	0.0	0.0	0.0	0.0	0.2	0.0	0.2	0.0	0.0
T/SN		0.0	0.0	0.0	0.0	0.0	0.0	0.0	0.0	0.0	0.0	0.0	0.0	0.0	0.0	0.0	0.0
TR		3.7	5.3	21.1	30.6	17.2	2.7	3.9	4.1	4.4	3.9	2.9	15.2	31.9	20.6	6.4	4.6
T/FX	CCF	0.0	0.3	0.0	0.0	11.5	1.1	0.2	0.2	0.0	0.2	0.5	10.5	0.0	0.0	0.7	0.0
	CCRF	0.0	0.3	0.0	0.0	2.9	0.2	0.0	0.0	0.0	0.0	0.2	4.0	0.0	0.0	0.2	0.0
	RF	0.0	0.2	0.2	0.2	0.2	1.3	1.9	2.4	2.9	1.4	2.9	0.4	0.3	0.0	0.2	0.0
	UCF	0.0	0.0	0.0	0.0	0.2	0.2	0.0	0.0	0.0	0.0	0.0	0.0	0.0	0.0	0.0	0.0

C, canine teeth; I, incisor teeth; M, molar teeth; P, premolar teeth; S, feline chronic gingivostomatitis (FCGS) only; SP, FCGS and periodontitis; SPTR, FCGS and periodontitis and tooth resorption; SPIRR, FCGS and periodontitis and inflammatory root resorption; CCF, complicated crown fracture; CCRF, complicated crown-root fracture; RF, root fracture; UCF, uncomplicated crown fracture.

**Table 4:** Prevalence of oral diseases in each tooth among mandibular extracted teeth.

Categories		Prevalence of oral diseases (%)													
		Right mandible							Left mandible						
		M1	P4	P3	C	I3	I2	I1	I1	I2	I3	C	P3	P4	M1
Number of extracted teeth		621	570	949	387	382	373	392	389	366	370	373	937	572	640
FN		0.0	0.0	0.0	0.0	0.0	0.0	0.0	0.0	0.0	0.0	0.0	0.0	0.0	0.0
MAL		0.3	0.0	0.0	1.6	0.3	0.0	0.0	0.0	0.0	0.0	1.3	0.0	0.0	0.3
OT		0.2	0.2	0.0	0.5	0.3	0.3	0.5	0.3	0.3	0.3	0.3	0.0	0.0	0.0
P		26.2	28.4	12.3	16.0	48.7	42.9	41.6	42.9	45.4	49.5	19.3	12.7	29.9	25.0
PA		2.1	3.5	1.6	3.4	0.5	0.3	0.3	0.0	0.0	0.3	4.3	1.6	3.3	2.3
PATR		0.2	0.4	0.1	0.5	0.0	0.0	0.0	0.0	0.0	0.0	0.0	0.1	0.3	0.2
PAIRR		1.3	0.2	0.0	0.8	0.0	0.0	0.0	0.0	0.0	0.0	0.0	0.0	0.3	0.6
PTR		11.8	10.5	14.8	7.5	0.5	0.5	0.5	0.3	0.3	0.5	6.4	14.2	10.5	12.0
PIRR		5.0	0.9	0.7	0.8	0.0	0.0	0.0	0.0	0.0	0.0	0.3	0.6	1.2	6.4
RTR		5.3	3.2	3.7	1.8	9.7	18.0	21.7	20.1	16.1	6.5	2.1	3.8	3.1	5.9
FCGS	S	5.5	11.4	5.5	10.6	11.3	11.0	9.9	9.5	9.6	11.6	10.5	5.7	11.7	4.8
	SP	22.1	24.7	11.5	23.5	27.2	24.1	20.2	21.1	24.9	27.8	24.1	11.0	23.4	21.3
	SPTR	4.2	3.0	3.9	2.1	0.0	0.3	0.0	0.3	0.5	0.5	2.1	4.2	3.5	4.4
	SPIRR	3.2	0.4	0.3	0.0	0.0	0.0	0.0	0.3	0.0	0.0	0.3	0.3	0.7	3.1
T/SN		0.0	0.9	0.2	0.0	0.0	0.0	0.0	0.0	0.0	0.0	0.0	0.2	0.3	0.0
TR		12.4	12.1	45.2	25.6	1.3	1.6	2.8	2.3	2.2	1.9	24.1	45.4	11.2	13.0
T/FX	CC	0.2	0.2	0.0	4.4	0.0	0.0	0.0	0.0	0.0	0.0	3.5	0.0	0.2	0.3
	CR	0.0	0.0	0.0	0.5	0.0	0.0	0.0	0.0	0.0	0.0	1.3	0.0	0.0	0.0
	RF	0.2	0.0	0.2	0.5	0.3	1.1	2.6	3.1	0.8	0.8	0.0	0.2	0.2	0.3
	UC	0.0	0.2	0.0	0.0	0.0	0.0	0.0	0.0	0.0	0.3	0.0	0.0	0.0	0.0

C, canine teeth; I, incisor teeth; M, molar teeth; P, premolar teeth; S, feline chronic gingivostomatitis (FCGS) only; SP, FCGS and periodontitis; SPTRF, FCGS and periodontitis and tooth resorption; SPIRR, FCGS and periodontitis and inflammatory root resorption; CCF, complicated crown fracture; CCRF, complicated crown-root fracture; RF, root fracture; UCF, uncomplicated crown fracture.

### 3.2 Distribution of extracted teeth due to periodontitis in 1,580 cats

#### 3.2.1 Number of extracted teeth due to periodontitis by sex, age, and breed

**Figure 4:**
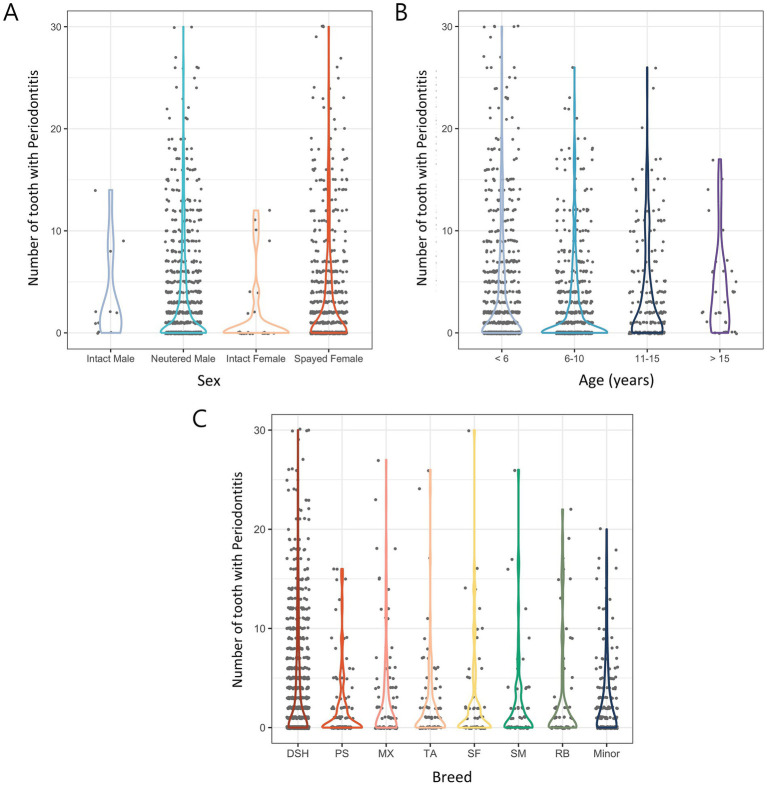
Number of extracted teeth due to periodontitis in 1,580 cats according to various categories. **(A)** Distribution of sex. **(B)** Distribution of age. **(C)** Distribution of breed. DSH, Domestic shorthair; PS, Persian; MX, Mixed; TA, Turkish Angora; SF, Scottish Fold; SM, Siamese; RB, Russian Blue.

The average number of teeth extracted per cat was highest in intact males (3.55), followed by spayed females (3.43), neutered males (3.28), and intact females (1.86). However, these differences were not statistically significant (*p* = 0.095; Figure 4A).

In a comparison based on age, Group 4 (> 15 years) had the most extracted teeth at 4.33, followed by Group 1 (< 6 years), Group 3 (11–15 years), and Group 2 (6–10 years) at 3.77, 3.59, and 2.61, respectively. Statistical analysis showed that cats older than 10 had significantly more extracted teeth than younger cats (*p* < 0.01) (Figure 4B).

In the comparison based on breeds, domestic shorthairs had the most extracted teeth at 3.8, followed by Russian Blues (3.25), mixed breeds (2.96), Scottish Folds (2.85), Siamese (2.5), Turkish Angoras (2.45), and Persians (2.05). In minor breeds, the number of extracted teeth was 2.79. Statistical analysis revealed no significant difference in the number of extracted teeth by breed (*p* = 0.076; Figure 4C).

#### 3.2.2 Proportion of extracted teeth due to periodontitis by tooth position

**Figure 5:**
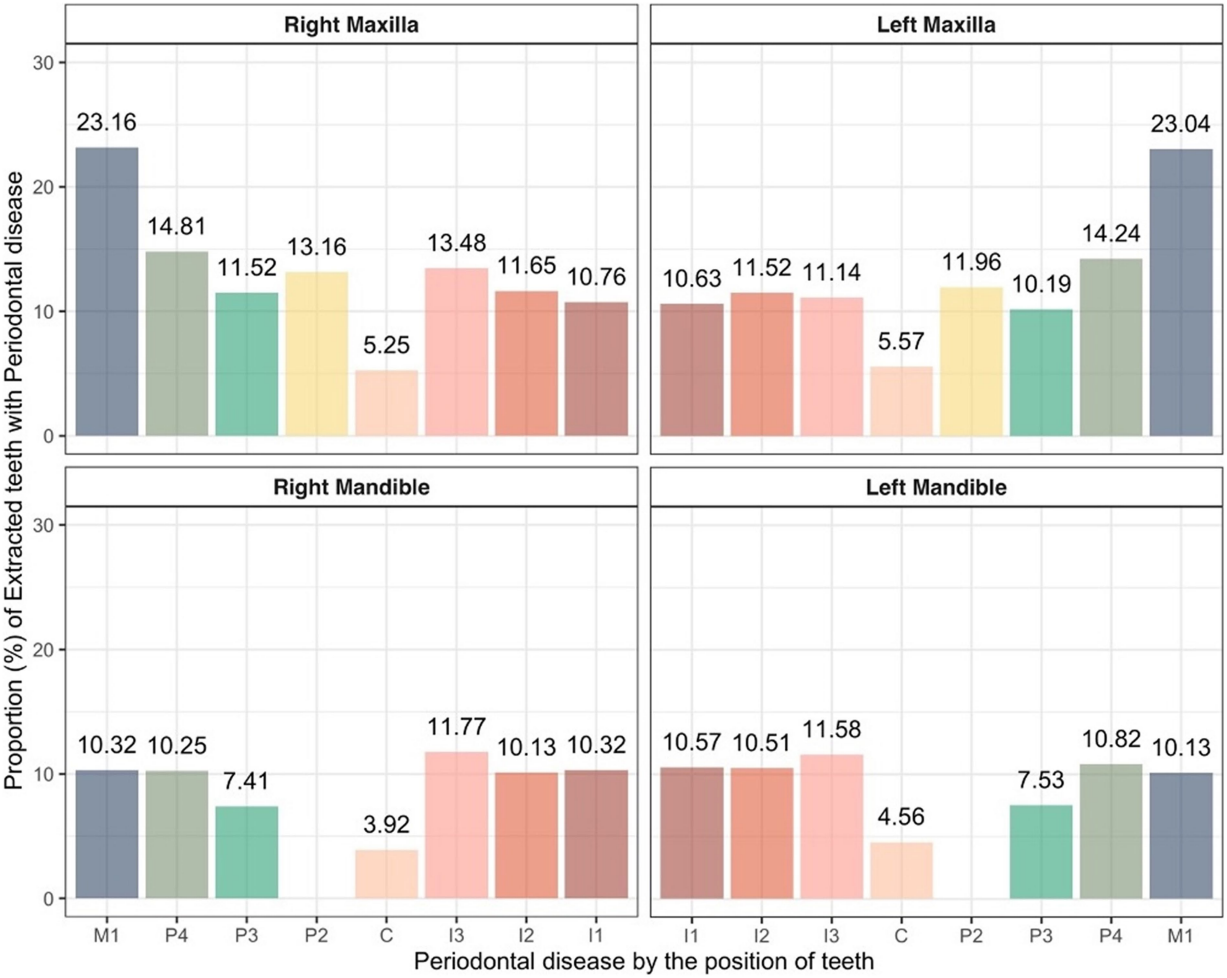
Proportion of extracted teeth due to periodontitis in cats based on tooth position. C, canine teeth; I, incisor teeth; M, molar teeth; P, premolar teeth.

The proportion of extracted teeth in both the maxilla and mandible showed similar trends on the left and right sides. In the maxilla, the highest extraction rates were of the right and left first molar teeth at 23.16 and 23.04%, respectively, and the lowest extraction rates were of the right and left canine teeth at 5.25 and 5.57%, respectively. In the mandible, the highest extraction rates were of the right and left third incisor teeth at 11.77% and 11.58, respectively, and as in the maxilla, the lowest extraction rates were of the right and left canine teeth at 3.92 and 4.56%, respectively (Figure 5).

### 3.3 Distribution of extracted teeth due to FCGS in 1,580 cats

#### 3.3.1 Number of extracted teeth due to FCGS by sex, age, and breed

**Figure 6:**
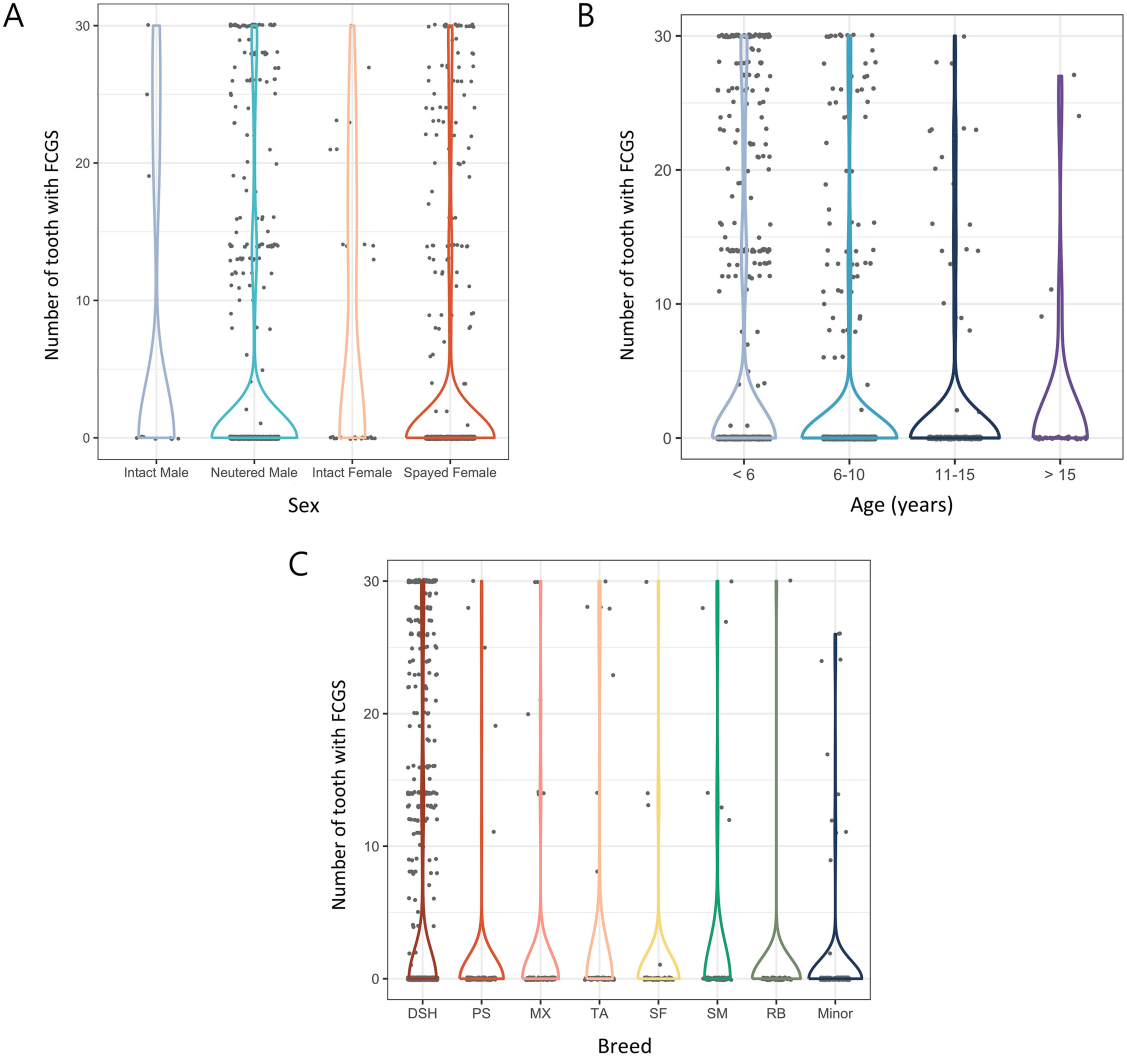
Number of extracted teeth due to FCGS in 1,580 cats by various categories. **(A)** Distribution of sex. **(B)** Distribution of age. **(C)** Distribution of breed. DSH, Domestic shorthair; PS, Persian; MX, Mixed; TA, Turkish Angora; SF, Scottish Fold; SM, Siamese; RB, Russian Blue.

In the sex comparison, intact females had most extracted teeth at 7.38, followed by intact males, neutered males, and spayed females at 6.73, 3.27, and 2.92, respectively. Statistical analysis showed no significant differences between males and females regardless of neutering status. However, intact females had significantly more extracted teeth than spayed females (*p* < 0.01) (Figure 6A).

In the age-based comparison, Group 1 (< 6 years) had the most extracted teeth at 4.49, followed by Group 2 (2.17), Group 4 (2.15), and Group 3 (1.85). In general, the number of extracted teeth decreased with increasing age. As a result of the statistical analysis, the number of extracted teeth was significantly higher in cats younger than 6 years than in cats of other ages (*p* < 0.001) (Figure 6B).

In a comparison among breeds, the number of extracted teeth was the highest in domestic shorthairs (4.66), followed by Turkish Angoras (2.2), Siamese (2.07), mixed breeds (1.4), Persians (0.97), Scottish Folds (0.94), and Russian Blues (0.51). In minor breeds, the number of extracted teeth was 0.97. Although overall breed differences were not significant, domestic shorthairs had significantly more extractions than other breeds (*p* < 0.05) (Figure 6C).

#### 3.3.2 Proportion of extracted teeth due to FCGS by tooth position

**Figure 7:**
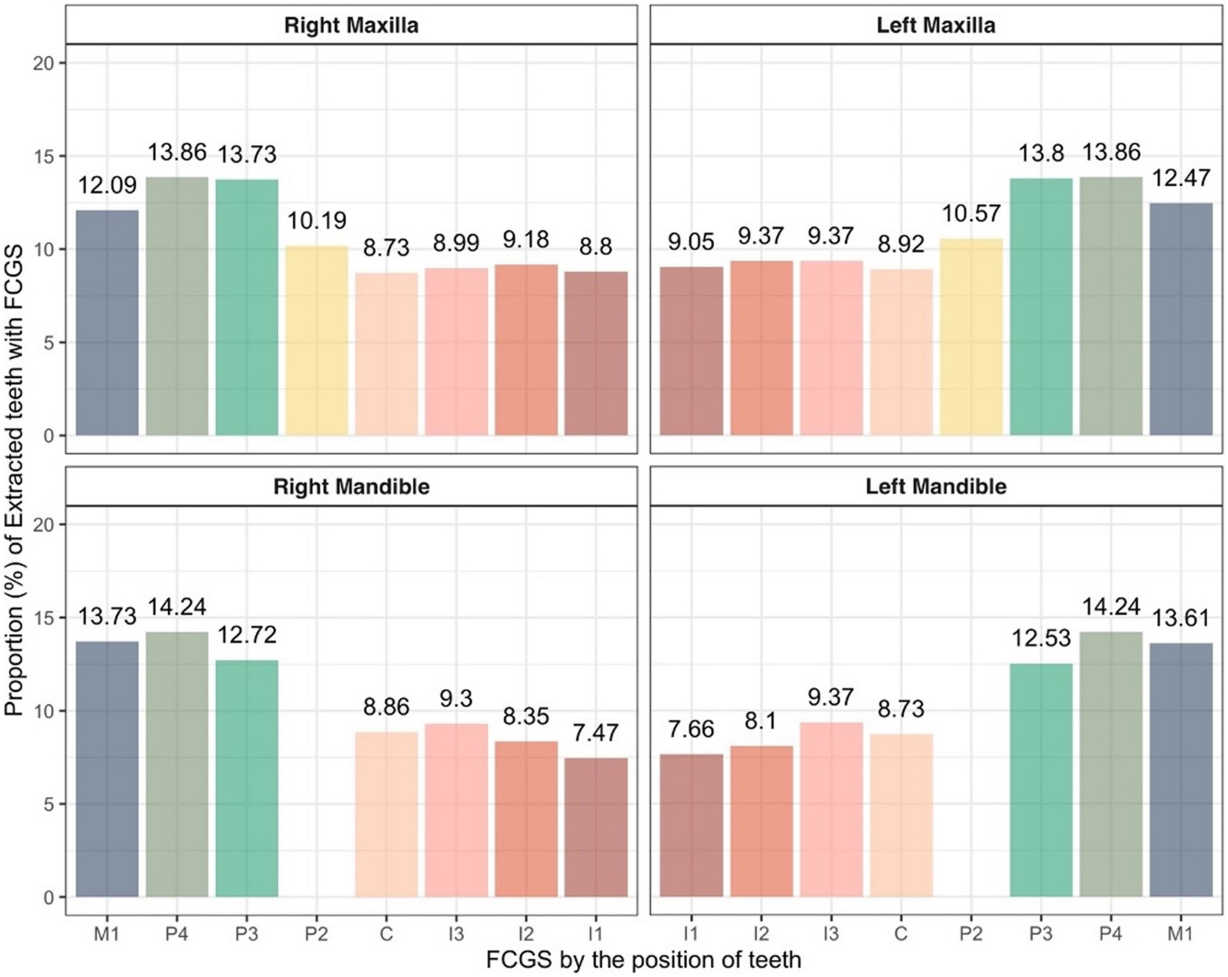
Proportion of extracted teeth due to FCGS in cats based on tooth position. C, canine teeth; I, incisor teeth; M, molar teeth; P, premolar teeth.

When analyzing the extracted teeth due to FCGS by tooth position, similar trends were observed in both the maxilla and mandible regardless of the affected side. In the maxilla, the right and left fourth premolar teeth showed the highest extraction rates at 13.86 and 13.86%, respectively, and the right and left canine teeth showed the lowest extraction rates at 8.73 and 8.92%, respectively. In the mandible, the right and left fourth premolar teeth showed the highest extraction rates at 14.24 and 14.24%, respectively, and the right and left canine teeth showed the lowest extraction rates at 8.86 and 8.73%, respectively. The statistical analysis results showed no significant differences in tooth position (Figure 7).

### 3.4 Distribution of extracted teeth due to TR in 1,580 cats

#### 3.4.1 Number of extracted teeth due to TR by sex, age, and breed

**Figure 8:**
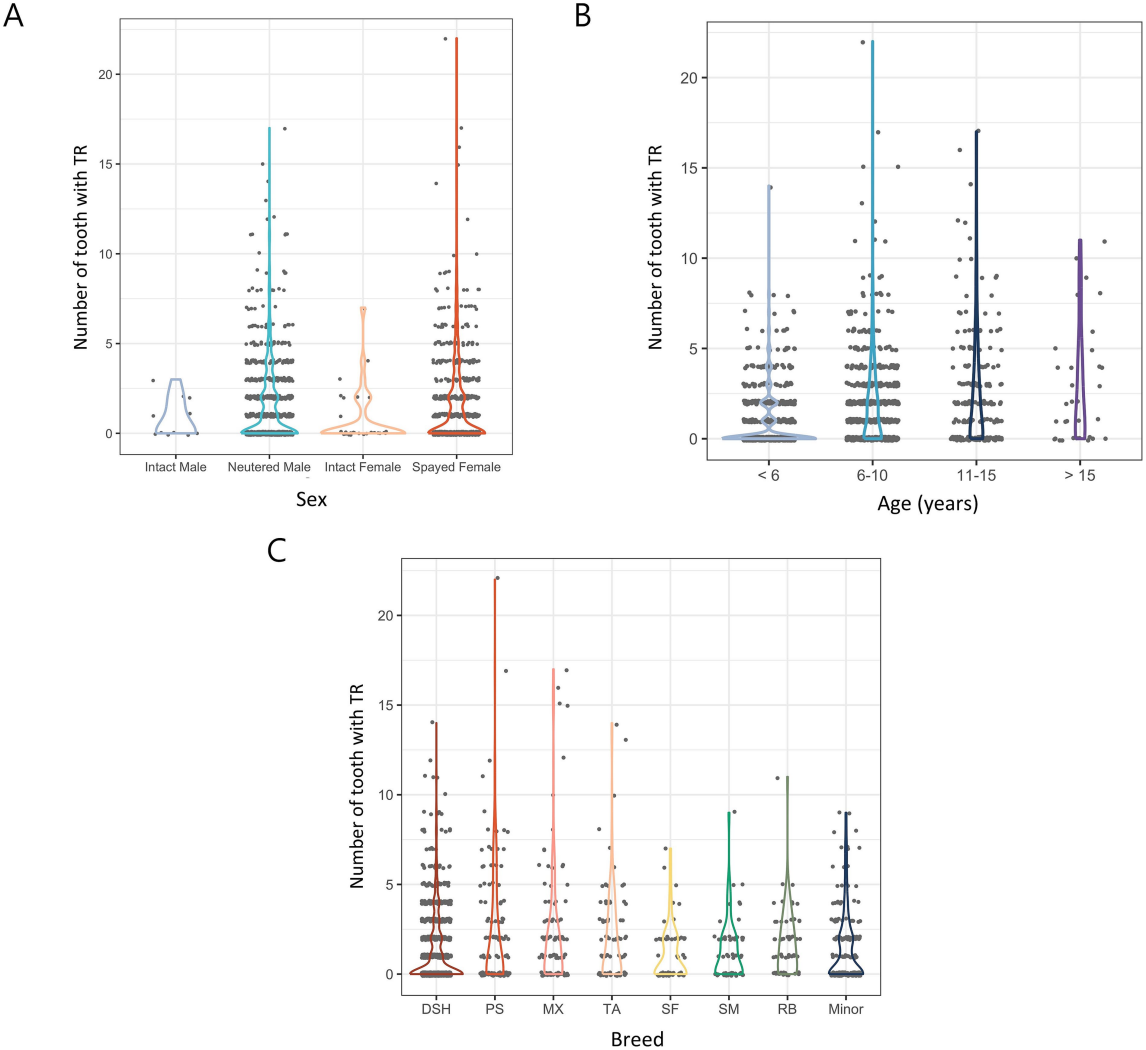
Number of extracted teeth due to TR in 1,580 cats by various categories. **(A)** Distribution of sex. **(B)** Distribution of age. **(C)** Distribution of breed. DSH, Domestic shorthair; PS, Persian; MX, Mixed; TA, Turkish Angora; SF, Scottish Fold; SM, Siamese; RB, Russian Blue.

Neutered males and spayed females had more teeth extracted (1.54 and 1.52, respectively), while intact males and intact females had fewer teeth extracted (0.82 and 0.79, respectively). There was no significant difference in the number of extracted teeth between sexes but neutered/spayed animals tended to have more extractions than intact animals. However, statistical analysis showed no significant difference (*p* = 0.127; Figure 8A).

In comparison based on age, Group 4 (> 15 years) had the highest number of extracted teeth (2.88), followed by Group 3 (2.43), Group 2 (1.99), and Group 1 (0.81). The number of extractions increased with age, and statistical analysis showed that cats younger than 6 years had significantly fewer TR-related extractions than those in older age groups (*p* < 0.001) (Figure 8B).

In a comparison between breeds, mixed breeds had the most extracted teeth (2.49), followed by Persians (2.44), Turkish Angoras (2.1), Russian Blues (1.71), Siamese (1.27), domestic shorthairs (1.27), and Scottish Folds (1.08). In minor breeds, the number of extracted teeth was 1.49. Statistical analysis showed that mixed breeds, Persians, Turkish Angoras, and Russian Blues had significantly more extractions due to TR than other breeds (*p* < 0.05) (Figure 8C).

#### 3.4.2 Proportion of extracted teeth due to TR by tooth position

**Figure 9:**
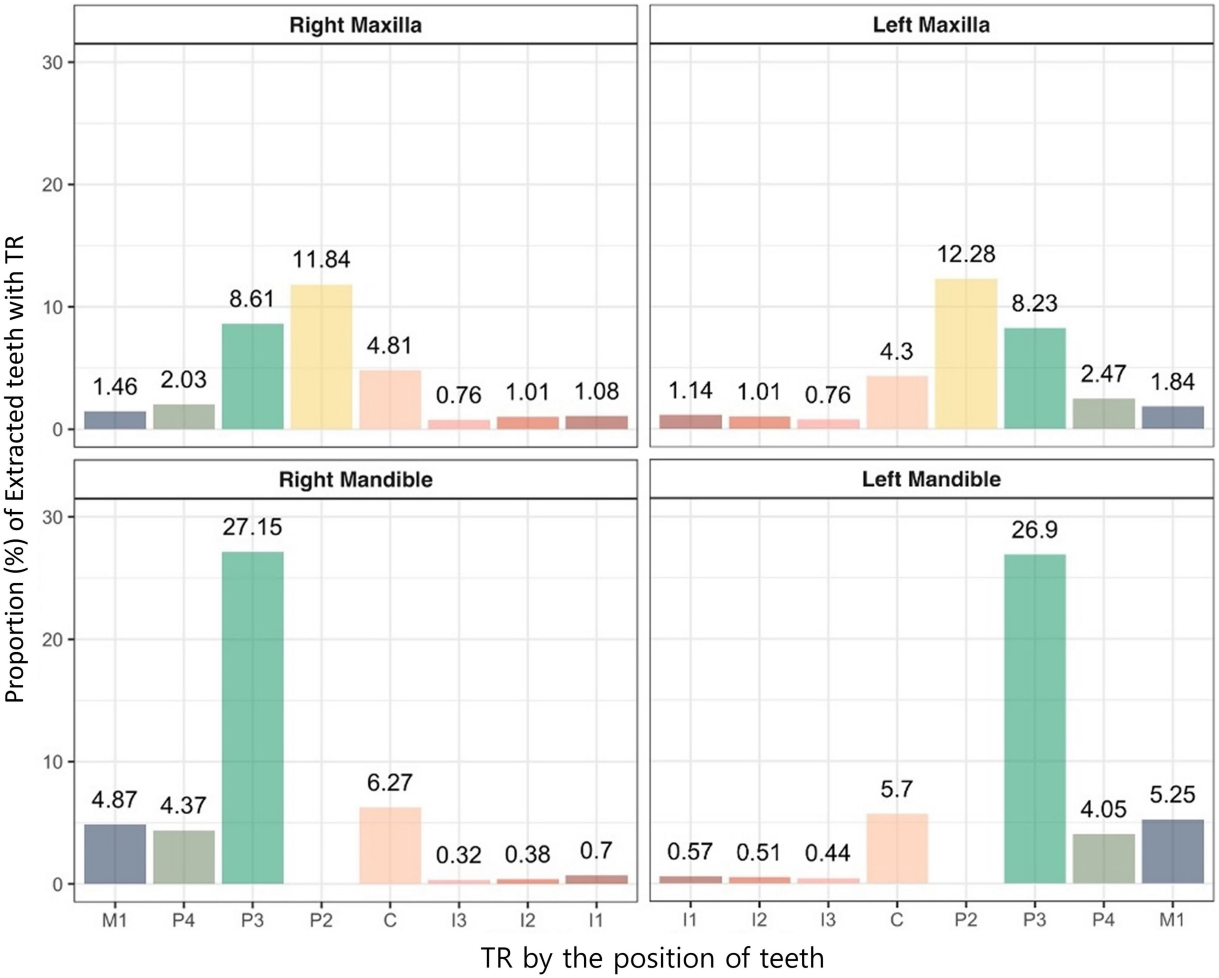
Proportion of extracted teeth due to TR in cats based on tooth position. C, canine teeth; I, incisor teeth; M, molar teeth; P, premolar teeth.

The extracted teeth due to TR were analyzed according to tooth position, and similar trends were observed in both the maxilla and the mandible regardless of the affected side. In the maxilla, the left and right second premolar teeth showed the highest extraction rates at 11.84 and 12.28%, respectively, and the left and right third incisor teeth showed the lowest extraction rates at 0.76 and 0.76%, respectively. In the mandible, the left and right third premolar teeth showed the highest extraction rates at 27.15 and 26.9%, respectively, and the left and right third incisor teeth showed the lowest extraction rates at 0.32 and 0.44%, respectively. In general, incisor teeth showed a low extraction rate, and premolar teeth showed a high extraction rate (Figure 9).

## 4 Discussion

This study identified periodontitis, TR, and FCGS as the most common reasons for tooth extraction in cats. Some of these conditions varied based on age, breed, or tooth position, emphasizing the need for individualized approaches to feline oral health. The study also aimed to identify the unique clinical characteristics of feline oral diseases, particularly in comparison to those seen in dogs. Most cats in this study were under 10 years of age and had been neutered or spayed. In addition, the predominant breeds matched those reported as the most popular in previous studies and statistical reports (24, 25).

Periodontitis was the most common cause of tooth extraction, followed by FCGS and TR. Other diseases were observed at relatively very low rates. A notable difference from previous studies on dogs (14) is that, while periodontitis accounts for more than half of extractions in dogs, its extraction rate was comparatively lower in cats. This difference can be attributed to feline-specific diseases such as FCGS and TR, which are uncommon in dogs. These findings highlight the need for a comprehensive understanding and detailed analysis of feline-specific oral diseases beyond periodontitis alone.

Analysis of periodontitis by sex showed that intact females showed a lower rate of tooth extraction due to periodontitis than other groups. However, the small sample size of intact cats made it challenging to establish a direct correlation between neutering and periodontitis-related extractions. Among neutered/spayed cats, extraction rates were similar regardless of sex, suggesting that sex does not significantly influence periodontitis-related extractions. Then, the extraction rate due to periodontitis was notably higher in older cats (>10 years) than in younger cats. Older animals often experience immunosenescences, which may reduce their ability to mount effective immune responses against oral pathogens. This may contribute to the increased incidence or severity of periodontitis in elderly cats (26). Additionally, older feline patients are often less likely to receive professional dental cleanings under anesthesia. This is often due to elevated anesthetic risks associated with systemic diseases, such as cardiovascular, renal, hepatic, endocrine, or neurological conditions, which can make owners reluctant to pursue elective procedures. As a result, periodontitis may progress unchecked, eventually requiring tooth extraction. This increased prevalence subsequently leads to a higher rate of tooth extractions in elderly cats. In the breed comparison, there was no significant difference in the number of teeth extracted due to periodontitis among breeds.

In the analysis by tooth location, periodontitis exhibited the highest extraction rates in the incisor teeth and molar teeth of the maxilla, while lower rate was observed in the canine teeth. Similarly, the incisor teeth had the highest extraction rates, while the canine and third premolar teeth showed relatively low rates in the mandible. The incisor teeth, located at the rostral part of the oral cavity, are more accessible for visual inspection and home care, such as tooth brushing, making them relatively easier for owners to manage. However, they have small, conical roots and less supporting tissue than other teeth. These anatomical features contribute to their increased susceptibility to tooth mobility and subsequent extraction in cases of advanced periodontitis. The last molar teeth in the maxilla, which are located at the innermost part of the oral cavity, are challenging to manage and are often prone to advanced disease due to limited visibility and accessibility. Similar to incisor teeth, these teeth are small, lack substantial supporting tissue, and are prone to tooth mobility, which may result in increased extraction rates. Key factors influencing extraction decisions include the degree of alveolar bone loss and tooth mobility (19–21). Therefore, the incisor teeth and the last molar teeth of the maxilla are more likely to experience tooth mobility than other teeth because of their smaller size. On the other hand, the canine teeth are the longest teeth in the maxilla, and they have long roots and robust supporting tissues, and they are the most easily observed during oral examination, making them relatively easy to manage. A similar trend has been observed in dogs in previous studies (14). Furthermore, the most common extractions related to periodontitis with alveolar bone expansion (ABE) were in the canine teeth. ABE is a common cause of canine tooth loss in cats; however, it is not associated with horizontal alveolar bone loss or tooth resorption patterns. Instead, ABE is characterized by a pattern of vertical alveolar bone loss (27).

Next, this study conducted a detailed analysis of FCGS, a condition that is commonly encountered in feline dentistry for which tooth extraction is often the primary treatment option (28, 29). In our results, more teeth were extracted due to FCGS in intact cats. However, the small sample size of the intact group limits the ability to draw definitive conclusions regarding the correlation between neutering status and FCGS-related extractions. Among spayed females and neutered males, the number of extracted teeth was nearly identical, further confirming that the effect of sex on FCGS-related tooth extractions is relatively minor. In addition, more extractions were observed in cats younger than 10 years of age. This aligns with the findings of previous studies indicating that FCGS commonly occurs between 3 and 14 years of age, with a median onset around 6 years (30, 31). Furthermore, one study reported that the prevalence of FCGS tends to decrease after 7 years of age (32). This age distribution highlights the need for early identification and management of FCGS in younger cats to reduce its impact on oral health and quality of life. In comparison by breed, domestic shorthair cats showed the most tooth extractions. Domestic breeds have adapted to living on their own in rural and urban areas and account for most free-roaming cats (33). Therefore, the number of extractions due to FCGS may be high in domestic shorthair cats adopted from free-roaming populations, as these cats may have been exposed to factors such as feline calicivirus infection, lack of early vaccination, or high environmental stress, which are known to be associated with the development of FCGS (34, 35).

Similar tooth extraction rates due to FCGS were observed across all teeth when compared by tooth position. Although previous studies have not directly linked viruses such as feline calicivirus (FCV), feline leukemia virus (FeLV), and feline immunodeficiency virus (FIV) to the development of FCGS, some have reported a relationship between FCV and worsening of clinical signs (13, 36). Consequently, the prevailing hypothesis suggests that FCGS is immune-mediated and associated with chronic viral infections (13, 37). Therefore, FCGS is a disease that affects the entire oral cavity rather than occurring frequently in specific teeth, and its primary treatment consists of full-mouth or partial-mouth extraction, determined by the distribution and severity of oral inflammation (38). A detailed analysis of extracted teeth due to FCGS revealed that more than half of these teeth also exhibited periodontitis. This finding supports the previous studies about a potential relationship between FCGS and periodontitis (39, 40).

There was difficulty establishing a direct relationship between neutering status and extraction frequency for TR due to the small sample size of intact cats. In addition, the extraction rates were similar between sexes, suggesting that sex did not significantly affect extraction due to TR, which is consistent with the results reported in previous studies (41–43). Previous research has indicated that TR typically occurs in cats older than 6 years of age (10, 41–44). Some studies have suggested that surface root cementum resorption occurs in all cats, and TR occurs when the lesion fails to heal (45). Therefore, the healing ability of a cat may indirectly affect the occurrence of TR. In older cats, the healing ability of the body is relatively reduced (26), which may interfere with the recovery of microscopic tooth resorption, potentially causing TR. Therefore, our results reinforce that TR is an age-dependent condition, likely reflecting its chronic and cumulative nature, which leads to a higher prevalence and severity of lesions in older cats. According to our study, the number of extracted teeth due to TR was significantly higher in mixed breeds, Persians, Turkish Angoras, and Russian Blues than in other breeds in the breed comparison. The exact pathogenesis of TR has not been identified, and the prevalent breeds in each study are slightly different (41–43). Therefore, while our study found higher TR prevalence in certain breeds (e.g., mixed breeds, Persians, Turkish Angoras, and Russian Blues), inconsistencies in the prevalent breeds reported across previous studies suggest that environmental factors may also play a significant role alongside potential genetic influences.

Finally, this study analyzed the location distribution of teeth extracted due to tooth resorption in cats. Previous studies have identified the mandibular third premolar teeth as the most frequently affected by TR (10, 44). One study also reported that radiographic examination of these teeth may allow for rapid screening of TR in cats (46). This study revealed significantly higher extraction rates for the more caudal teeth, particularly the maxillary second and third premolar teeth and the mandibular third premolar teeth. These findings are consistent with those of previous studies, however, the exact mechanism underlying the high susceptibility of the mandibular third premolars remain unclear.

This study systematically analyzed common oral diseases in cats and the characteristics associated with tooth extraction, underscoring the importance of feline oral health management. Periodontitis, FCGS, and TR were identified as the primary conditions leading to tooth extraction. These findings highlight the need to develop tailored treatment and management strategies for feline oral diseases. In future studies, it will be important to deeply explore the etiology of and preventive strategies for oral diseases in cats through more diverse samples and long-term follow-up observations. This study had several limitations. First, although the retrospective nature of the study inherently poses challenges in data consistency, we attempted to minimize such limitations by including only those cats with complete and standardized medical records, full-mouth radiographs, and dental chart documentation. Second, a potential selection bias could have occurred, as the two participating hospitals were dental specialty practices. Therefore, cats with more severe clinical signs or owners more invested in veterinary care may have been overrepresented. Third, the sample was predominantly composed of domestic shorthair cats, limiting breed-specific comparisons. Future studies should include more diverse and balanced populations and utilize standardized criteria to strengthen the generalizability of findings.

## 5 Conclusion

This study thoroughly investigated the causes of tooth extractions in cats, revealing the differences between oral disorders that affect dogs and cats. Although the most common cause was found to be periodontitis, feline-specific disorders such as FCGS and TR also contributed significantly to tooth extractions. In addition, all three diseases showed differences in their prevalence with age. These results highlight the importance of tailoring oral health management strategies—such as daily tooth brushing, regular dental examinations, and professional dental cleanings—combined with early diagnosis and timely treatment, which are essential for managing age-related dental diseases in cats. No significant differences in dental extraction rates were observed by sex or neutering status, but the small sample size of intact cats precludes definitive conclusions. Furthermore, as most of the sampled population consisted of domestic shorthair cats, the findings may not fully represent other breeds. Given that the characteristics of domestic cats can vary by region, caution is warranted when generalizing these results to other populations. Future studies should incorporate a broader range of breeds, larger sample sizes, and long-term follow-up could provide more comprehensive insights into feline oral health and support the development of improved preventive care and treatment approaches.

## Data Availability

The original contributions presented in the study are included in the article/supplementary material, further inquiries can be directed to the corresponding author.

## References

[1] Lund EM Armstrong PJ Kirk CA Kolar LM Klausner JS. Health status and population characteristics of dogs and cats examined at private veterinary practices in the United States. J Am Vet Med Assoc. (1999) 214:1336–41. doi: 10.2460/javma.1999.214.09.1336, PMID: 10319174

[2] Girard N Servet E Biourge V Hennet P. Periodontal health status in a colony of 109 cats. J Vet Dent. (2009) 26:147–55. doi: 10.1177/089875640902600301 19950514

[3] O’Neill DG Blenkarn A Brodbelt DC Church DB Freeman A. Periodontal disease in cats under primary veterinary care in the UK: frequency and risk factors. J Feline Med Surg. (2023) 25:1098612X231158154. doi: 10.1177/1098612X231158154, PMID: 36912667 PMC10812011

[4] O'Neill D Mitchell C Humphrey J Church D Brodbelt D Pegram C. Epidemiology of periodontal disease in dogs in the UK primary-care veterinary setting. J Small Anim Pract. (2021) 62:1051–61. doi: 10.1111/jsap.13405, PMID: 34374104 PMC9291557

[5] Verstraete FJ Kass PH Terpak CH. Diagnostic value of full-mouth radiography in cats. Am J Vet Res. (1998) 59:692. doi: 10.2460/ajvr.1998.59.06.692 9622736

[6] Palmeira I Fonseca MJ Lafont-Lecuelle C Pageat P Cozzi A Asproni P. Dental pain in cats: a prospective 6-month study. J Vet Dent. (2022) 39:369–75. doi: 10.1177/08987564221103142, PMID: 35603830 PMC9638711

[7] DVM360. (2010). Extractions in cats: indications, techniques and complications (proceedings). Available online at: https://www.dvm360.com/view/extractions-cats-indications-techniques-and-complications-proceedings (accessed December 7, 2024).

[8] Reiter AM Johnston N Anderson JG Soltero-Rivera MM Lobprise HB. Domestic feline oral and dental diseases In: Lobprise HB Dodd JRB, editors. Wiggs's veterinary dentistry: Principles and practice. 2nd ed. Hoboken, NJ: John Wiley & Sons, Inc. (2019). 439–61.

[9] Lommer MJ Verstraete FJ. Prevalence of odontoclastic resorption lesions and periapical radiographic lucencies in cats: 265 cases (1995–1998). J Am Vet Med Assoc. (2000) 217:1866–9. doi: 10.2460/javma.2000.217.1866 11132894

[10] Gorrel C. Tooth resorption in cats: pathophysiology and treatment options. J Feline Med Surg. (2015) 17:37–43. doi: 10.1177/1098612X14560098, PMID: 25527492 PMC11383098

[11] Soltero-Rivera M Shaw C Arzi B Lommer M Weimer BC. Feline chronic gingivostomatitis diagnosis and treatment through transcriptomic insights. Pathogens. (2024) 13:192. doi: 10.3390/pathogens13030192, PMID: 38535535 PMC10974286

[12] Papadimitriou S Kouki M. The role of dental extractions in feline chronic gingivostomatitis (Fcgs). EC Vet Sci. (2016) 2:170

[13] Soltero-Rivera M Goldschmidt S Arzi B. Feline chronic gingivostomatitis current concepts in clinical management. J Feline Med Surg. (2023) 25:1098612X231186834. doi: 10.1177/1098612X231186834, PMID: 37548475 PMC10811996

[14] Kim C-G Kwon D Lee K Kim SE Jo HM. Prevalence of reasons for tooth extraction in small-and medium-breed dogs. Animals. (2025) 15:224. doi: 10.3390/ani15020224, PMID: 39858224 PMC11758340

[15] American Veterinary Dental College. (2025). AVDC nomenclature. Teeth abnormalities and related procedures. Available online at: https://avdc.org/avdc-nomenclature/ (accessed April 29, 2025).

[16] Logan E Boyce E. Oral health assessment in dogs: parameters and methods. J Vet Dent. (1994) 11:58–63. 9693614

[17] Wolf HF Rateitschak-Pluss EM Rateitschak KH Hassell TM. Color atlas of dental medicine: Periodontology. 3rd ed. Stuttgart: Georg Thieme Verlag (2005). p. 79–84.

[18] American Veterinary Dental College. (2025). AVDC nomenclature. Periodontal anatomy and disease. Avaiable online at: https://avdc.org/avdc-nomenclature/ (accessed April 29, 2025).

[19] Lommer MJ Tsugawa AJ Verstraete FJM. Extraction of multirooted teeth in dogs In: Verstraete FJM Lommer MJ Arzi B, editors. Oral and maxillofacial surgery in dogs and cats. 2nd ed. St. Louis, MO: Elsevier (2020). 151–9.

[20] Lommer MJ. Special considerations in feline exodontics In: Verstraete FJM Lommer MJ Arzi B, editors. Oral and maxillofacial surgery in dogs and cats. 2nd ed. St. Louis, MO: Elsevier (2020). 160–72.

[21] Lommer MJ Verstraete FJM. Simple extraction of single-rooted teeth In: Verstraete FJM Lommer MJ Arzi B, editors. Oral and maxillofacial surgery in dogs and cats. 2nd ed. St. Louis, MO: Elsevier (2020). 136–41.

[22] Lommer MJ. Principles of exodontics In: Verstraete FJM Lommer MJ Arzi B, editors. Oral and maxillofacial surgery in dogs and cats. 2nd ed. St. Louis, MO: Elsevier (2020). 118–35.

[23] Kan-Rohrer KH Terpak CH Verstraete FJM. Instrumentation, aseptic technique, and patient preparation In: Verstraete FJM Lommer MJ Arzi B, editors. Oral and maxillofacial surgery in dogs and cats. 2nd ed. St. Louis, MO: Elsevier (2020). 65–78.

[24] Statista (2023). Most Popular Cat Breeds South Koreans in Based on ownership (2025). Avaialble online at: https://www.statista.com/statistics/960183/popular-breed-cat-south-korea/ (accessed January 10, 2025).

[25] Lee J Pak S-i Lee K Choi H Lee Y Park I. Feline demographics and disease distribution in the Republic of Korea. J Vet Clin. (2022) 39:217–25. doi: 10.17555/jvc.2022.39.5.217

[26] Day M. Ageing, immunosenescence and inflammageing in the dog and cat. J Comp Pathol. (2010) 142:S60–9. doi: 10.1016/j.jcpa.2009.10.011 20005526

[27] Peralta S Fiani N Scrivani PV. Prevalence, radiographic, and demographic features of buccal bone expansion in cats: a cross-sectional study at a referral institution. J Vet Dent. (2020) 37:66–70. doi: 10.1177/0898756420953581 32875972

[28] Rivas IL Soltero-Rivera M Vapniarsky N Arzi B. Stromal cell therapy in cats with feline chronic gingivostomatitis: current perspectives and future direction. J Feline Med Surg. (2023) 25:1098612X231185395. doi: 10.1177/1098612X231185395, PMID: 37548494 PMC10811994

[29] Arzi B Taechangam N Lommer MJ Walker NJ Loscar MR Borjesson DL. Stem cell therapy prior to full-mouth tooth extraction lacks substantial clinical efficacy in cats affected by chronic gingivostomatitis. J Feline Med Surg. (2021) 23:604–8. doi: 10.1177/1098612X20967172, PMID: 33118849 PMC10741287

[30] Healey KA Dawson S Burrow R Cripps P Gaskell CJ Hart CA. Prevalence of feline chronic gingivo-stomatitis in first opinion veterinary practice. J Feline Med Surg. (2007) 9:373–81. doi: 10.1016/j.jfms.2007.03.003, PMID: 17507275 PMC10832963

[31] Vapniarsky N Simpson DL Arzi B Taechangam N Walker NJ Garrity C. Histological, immunological, and genetic analysis of feline chronic gingivostomatitis. Front Vet Sci. (2020) 7:310. doi: 10.3389/fvets.2020.00310, PMID: 32582783 PMC7283503

[32] Clarke DE Caiafa A. Oral examination in the cat: a systematic approach. J Feline Med Surg. (2014) 16:873–86. doi: 10.1177/1098612X14552364, PMID: 25344458 PMC11044606

[33] Ogan CV Jurek R. Biology and ecology of feral, free-roaming, and stray cats. In: *Proceedings of the Mesocarnivores of northern California: biology, management, and survey techniques, workshop manual*; Alcata, CA: California North Coast Chapter (1997), pp. 87–92.

[34] Kim D-H Kwak H-H Woo H-M. Prevalence of feline chronic gingivostomatitis in feral cats and its risk factors. J Feline Med Surg. (2023) 25:1098612X221131453. doi: 10.1177/1098612X221131453, PMID: 36655688 PMC10812041

[35] Druet I Hennet P. Relationship between feline calicivirus load, oral lesions, and outcome in feline chronic gingivostomatitis (caudal stomatitis): retrospective study in 104 cats. Front Vet Sci. (2017) 4:209. doi: 10.3389/fvets.2017.00209, PMID: 29270412 PMC5724031

[36] Tenorio AP Franti CE Madewell BR Pedersen NC. Chronic Oral infections of cats and their relationship to persistent Oral carriage of feline Calici-, immunodeficiency, or leukemia viruses. Vet Immunol Immunopathol. (1991) 29:1–14. doi: 10.1016/0165-2427(91)90048-H, PMID: 1659031

[37] Arzi B Mills-Ko E Verstraete FJ Kol A Walker NJ Badgley MR. Therapeutic efficacy of fresh, autologous mesenchymal stem cells for severe refractory gingivostomatitis in cats. Stem Cells Transl Med. (2016) 5:75–86. doi: 10.5966/sctm.2015-0127, PMID: 26582907 PMC4704876

[38] Johnston N. An updated approach to chronic feline gingivitis stomatitis syndrome. Vet Pract. (2012) 44:34–8.

[39] Lyon KF. Gingivostomatitis. Vet Clin North Am Small Anim Pract. (2005) 35:891–911. doi: 10.1016/j.cvsm.2005.02.001, PMID: 15979518

[40] Farcas N Lommer MJ Kass PH Verstraete FJ. Dental radiographic findings in cats with chronic gingivostomatitis (2002–2012). J Am Vet Med Assoc. (2014) 244:339–45. doi: 10.2460/javma.244.3.339 24432966

[41] Girard N Servet E Biourge V Hennet P. Feline tooth resorption in a Colony of 109 cats. J Vet Dent. (2008) 25:166–74. doi: 10.1177/089875640802500302, PMID: 19025137

[42] Pistor P Janus I Janeczek M Dobrzyński M. Feline tooth resorption: a description of the severity of the disease in regard to animal’s age, sex, breed and clinical presentation. Animals. (2023) 13:2500. doi: 10.3390/ani13152500, PMID: 37570307 PMC10417119

[43] Vapalahti K Neittaanmäki H Lohi H Virtala A-M. A large case-control study indicates a breed-specific predisposition to feline tooth resorption. Vet J. (2024) 305:106133. doi: 10.1016/j.tvjl.2024.106133, PMID: 38740176

[44] Mestrinho LA Runhau J Bragança M Niza MM. Risk assessment of feline tooth resorption: a Portuguese clinical case control study. J Vet Dent. (2013) 30:78–83. doi: 10.1177/089875641303000202 24006716

[45] Gorrel C Larsson Å. Feline odontoclastic resorptive lesions: unveiling the early lesion. J Small Anim Pract. (2002) 43:482–8. doi: 10.1111/j.1748-5827.2002.tb00018.x 12463263

[46] Heaton M Wilkinson J Gorrel C Butterwick R. A rapid screening technique for feline odontoclastic resorptive lesions. J Small Anim Pract. (2004) 45:596–601. doi: 10.1111/j.1748-5827.2004.tb00181.x 15600270

